# COVID-19: The Case of Three Patients with the Same Diagnosis but Different Clinical and Laboratory Features

**DOI:** 10.1155/2020/9185041

**Published:** 2020-05-24

**Authors:** Muhammed Atere, Sukhdev Singh, Krisha Arora, Zohaib Khan, Lloyd Muzangwa, Urvi Bhavsar, Jay M. Nfonoyim

**Affiliations:** Richmond University Medical Center, 355 Bard Avenue, Staten Island, New York, NY 10310, USA

## Abstract

SARS-CoV-2 is an RNA virus that causes COVID-19, which has been responsible for the pandemic that was declared in early 2020. Its pathological effect is majorly in the respiratory tract, but its full pathogenicity remains a mystery. Symptoms associated with COVID-19 include fever, cough, and shortness of breath. Some patients develop other symptoms like diarrhea. However, it is possible for other organs to be affected including the central nervous system, liver, and blood cells. The purpose of this case series is to unravel other factors associated with this disease, so we report three cases of COVID-19 that were hospitalized during the pandemic.

## 1. Introduction

Many viruses may infect both the upper and lower respiratory tracts, but infection of the lower respiratory tract may lead to more complications. Influenza viruses are particularly notorious for infecting the respiratory system with peaks during the winter season, but another virus that also has a predilection for the respiratory system became a pandemic in early 2020. The virus, SARS-CoV-2, is an RNA virus belonging to the Coronaviridae family, and the associated disease is known as COVID-19 [1–3]. SARS-CoV-2 is a virulent zoonotic organism with significant mortality [1, 2, 4, 5]. It has been documented to be transmitted through the air and droplets from infected persons [1, 3, 4]. Clinical features include cough and fever [1]. Its unfamiliarity has made it difficult to tackle and prevent the continued spread. The purpose of this case report is to highlight salient points that may assist clinicians in recognizing, managing, and preventing this disease. Additionally, we plan to delineate other uncommon features that may develop in patients with the infection. Here, we report the cases of three hospitalized patients with positive laboratory results for COVID-19.

## 2. Case Reports

We describe three cases of COVID-19 while including only significant and specific information about the history, vital signs, examination, investigations, and treatment.

### 2.1. Case 1

**Figure 1:**
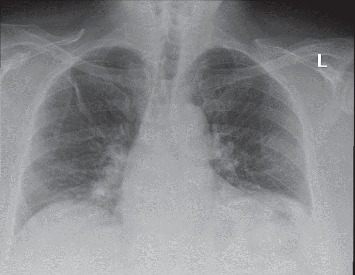
A chest X-ray with an anterior-posterior view: diagonal band-like density in the right upper lobe and linear densities at the bases.

A 67-year-old male with a past medical history of asthma and hypertension presented to the emergency room for evaluation of a 5-day history of fever, cough, chest pain, shortness of breath, and body aches (Table 1). The patient was seen at an urgent care center and was diagnosed with bronchitis, but his symptoms worsened. He also had a chest X-ray, which indicated pneumonia and was treated with Augmentin and doxycycline. He traveled recently to Aruba, but he denied sick contacts. The initial vitals showed a temperature of 97.9 Fahrenheit, pulse rate of 104 beats per minute, respiratory rate of 20 cycles per minute, blood pressure of 143/91 mmHg, and pulse oximetry of 97% on room air. Physical examination was unremarkable. Initial laboratory showed normal complete blood count but a white blood cell count of 4.1 k/*μ*L, platelets of 121 k/*μ*L, aspartate transaminase of 48 U/L, and alanine transaminase of 92 U/L. Urine was negative for *Legionella* and *Streptococcus pneumoniae* antigens. Chest X-ray demonstrated diagonal band-like density in the right upper lobe and linear densities at the bases (Figure 1). His oronasal swab was positive for SARS-CoV-2. He was started on ceftriaxone and azithromycin before he was transitioned to levofloxacin and hydroxychloroquine.

### 2.2. Case 2

A 46-year-old male with no significant past medical history presented to the emergency room because of an episode of seizures, cough, fever, vomiting, diarrhea, and syncope (Table 1). His symptoms started three days before presentation. His home temperature was 101.6 Fahrenheit, but it resolved with acetaminophen. On the day of presentation, he syncopized, vomited, and had one episode of seizures. He had an involuntary loss of feces, but he regained consciousness after 30 seconds. He also had a similar episode in the emergency room on the same day but for a duration of two minutes. He denied a headache, chest pain, shortness of breath, palpitations, abdominal pain, or runny nose. His initial vital signs revealed a temperature of 99.1 Fahrenheit peaking at 102.9 Fahrenheit the following day, pulse rate of 86 beats per minute, blood pressure of 130/90 mmHg, and pulse oximetry of 95% on two liters of intranasal oxygen. Physical examination was essentially unremarkable. The initial laboratory investigations revealed a normal complete blood cell count but sodium of 131 mmol/L, creatinine of 1.9 mg/dl, aspartate transaminase of 62 U/L, alanine transaminase of 73 U/L, and alkaline phosphatase of 225 U/L. A urine drug screen and urinalysis were insignificant. However, his creatine phosphokinase was elevated at 1427 U/L. A chest X-ray revealed grossly clear lungs. A head CT and MRI were unremarkable. A lumbar puncture for cerebrospinal fluid was temporarily deferred because of the lethality of the disease and its unlikeliness to change the management. Laboratory result was eventually positive for SARS-CoV-2. He was originally treated with vancomycin, ceftriaxone, and acyclovir before they were switched to azithromycin and hydroxychloroquine.

### 2.3. Case 3

**Figure 2:**
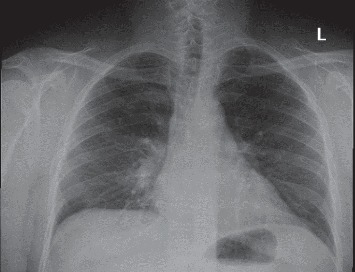
Chest X-ray with an anterior-posterior view: mild fullness in the right perihilar region.

A 43-year-old male with no significant past medical history presented to the emergency room with shortness of breath, cough, fever, chest pain, and headache for about one week (Table 1). He was taking doxycycline for pneumonia before presentation; however, his symptoms did not resolve. He denied sick contacts, recent travel, or being in contact with someone who traveled. He also denied vomiting, diarrhea, or abdominal pain. Initial vitals showed a temperature of 98.7 Fahrenheit but rising to 101.2 Fahrenheit, pulse rate of 76 beats per minute, respiratory rate of 18 cycles per minute, blood pressure of 121/75 mmHg, and pulse oximetry of 95% on room air. Physical examination revealed breath sounds that were equal bilaterally. The initial laboratory showed a white blood cell count of 2.1 k/*μ*L, hemoglobin of 12.8 g/dl, and platelets of 110 k/*μ*L, but a complete metabolic panel was normal. Laboratory investigation was also negative for HIV 1 and 2, influenza A and B, and respiratory syncytial virus. A chest X-ray demonstrated mild fullness in the right perihilar region (Figure 2.). He was diagnosed with COVID-19 and was started on levofloxacin but he received hydroxychloroquine for two days.

## 3. Discussion

Coronavirus is a part of the Coronaviridae family, and it is a nonsegmented, enveloped, positive-sense RNA virus [1, 2]. It is a virus with a high mutation rate and a genome-encoded exonuclease which causes the zoonotic viral pathogen to modify and potentially become more virulent [3]. Six identified species of coronavirus infect humans. Four of them (HCoV-229E, HCoV-OC43, HCoV-NL63, and HCoV-HKU1) cause common cold symptoms while the other two (SARS-CoV and MERS-CoV) are zoonotic beta coronaviruses that cause severe respiratory distress [1, 2, 4, 5]. SARS-CoV-2 is more like the zoonotic types in terms of severity and the organ involved, and all three of them have origins in bats [1, 2, 4, 5]. The genome sequence of SARS-CoV-2 shows the most similarities with SARS-CoV and SARS-like bat CoV [6]. They both bind to the angiotensin-converting enzyme-2 (ACE-2) receptor in human host cells [6].

Several of the first cases of pneumonia of unknown causes in Wuhan, China, were linked to the Huanan Seafood Market, which was closed as a result [1, 3]. COVID-19 was added to the Notifiable Communicable Disease list, and public health measures are in place to lessen the spread of the virus [3]. More than 50% of affected patients were males [7]. The total number of cases in the United States until May 8, 2020, is over 1.2 million and total death of more than 73,000 with an expected increase in new cases and mortality [5].

The exact method of transmission is unknown, but it has been labeled as an airborne virus [1]. Person-to-person transmission via droplets from coughing or sneezing or direct contact has been reported [3, 4, 8]. Respiratory droplets and direct contact with bodily fluids or contaminated surfaces have also been postulated [7, 9]. Since SARS-CoV-2 can be detected in urine, saliva, and the gastrointestinal tract, other forms of transmission are still under investigation [7, 10]. The Centers for Disease Control and Prevention (CDC) has recommended droplet, contact, and airborne precautions along with protection of the eyes for confirmed cases [10]. Tropism for nonrespiratory mucosa as seen in the eyelids has also been documented [9].

Most patients present with fever, cough, chills, myalgia, fatigue, and dyspnea but less commonly seen are sputum production, headache, nausea, vomiting, diarrhea, and hemoptysis [1, 7, 11]. Only a few cases are found to be associated with gastrointestinal symptoms like diarrhea [3]. Impaired immunity is observed with lymphopenia and elevated C-reactive protein [7, 8]. It has a wide spectrum of severity. Some patients may present with viral pneumonia, while others may not develop pneumonia at all [1, 7]. Asymptomatic people positive for COVID-19 are like reservoirs for the virus as they may present with mild or no symptoms but can increase the spread with high efficiency, despite conventional measures of protection [9, 11]. There may also be extended periods of shedding after recovery [9, 11]. The incubation period has been reported as ranging from two days to two weeks after exposure [4].

Investigations performed in suspected cases include specimens taken from the lower respiratory tract and detected using reverse transcriptase-polymerase chain reaction (RT-PCR) methods [1]. The extraction of nucleic acids from bronchoalveolar lavage fluid using a High Pure Viral Nucleic Acid Kit to test for viruses and bacteria by PCR is also another method of diagnosis [2]. Nasopharyngeal and oropharyngeal swabs with RT-PCR is another essential tool for diagnosis [10]. However, autopsy and biopsy would help to further understand the virus [1]. Many of the patients undergo imaging. Chest CT is more sensitive than X-ray in terms of identifying viral pneumonia [8]. Chest CT may also show consolidation or bilateral ground-glass opacities [3, 7]. However, chest CT may be unnecessary if the diagnosis is confirmed or the disease is highly suspected.

Complications of COVID-19 include acute respiratory distress syndrome (ARDS), acute cardiac injury, and secondary infections that require invasive mechanical ventilation [1, 7]. Worse outcomes are seen in patients who present with respiratory distress on admission [7]. Being a novel virus, there are no specific drugs against it. Empiric antibiotics along with oseltamivir, methylprednisolone, and oxygen support have been tried [1, 7]. Other medications tried are moxifloxacin, levofloxacin, nemonoxacin, linezolid, umifenovir, azithromycin and amoxicillin, cefepime, vancomycin, and intravenous remdesivir (developed against Ebola) [8, 10, 12]. A controlled trial of ritonavir-boosted lopinavir is in place for COVID-19, since there are no specific antivirals with proven effectiveness against the virus in humans [3]. Chloroquine decreased hospital stays and lessened the extent of pneumonia spread in patients with the disease [13]. Hydroxychloroquine has also been used, and it has a similar mechanism of action to chloroquine [13]. Remdesivir has been approved by the United States Food and Drug Administration for treating severe COVID-19 [12]. Other newer and experimental drugs are expected to be developed soon.

Our patients developed similar symptoms that have been reported, but one of them had a new-onset seizure, which may suggest that SARS-CoV-2 may possibly cause encephalitis. The sodium level, although mildly low, was considered less likely to induce a seizure. Two of our patients had thrombocytopenia on presentation while one of them had pancytopenia. Solitary thrombocytopenia and pancytopenia may be a laboratory feature of this virus. Among the three patients, two of them had mild transaminitis, which may indicate possible viral hepatitis in some patients. One of the patients was eventually discharged, but the other two remained hospitalized but clinically stable while receiving hydroxychloroquine.

Like other respiratory viral infections, preventing transmission is the hallmark to decrease the incidence. Frequent hand washing, avoidance of touching of eyes, nose, and mouth, covering of sneezes and coughs, and self-isolation are recommended [14]. Aggressive methods of protection (N-95 masks, goggles, and protective gowns) are necessary for healthcare workers with direct contact with patients with the virus [1, 7, 9].

## Figures and Tables

**Table 1:** Summary of cases.

Age	Sex	Past medical history	Symptoms and signs	Initial vitals	Physical examination	Positive labs	Chest X-ray	Treatment	Outcome during the composition of case series
67	M	Asthma, hypertension	Fever, cough, body ache, chest pain	Temperature 97.9 Fahrenheit, pulse of 104	Equal breath sounds	Platelets 121, aspartate transaminase 48, alanine transaminase 92	Diagonal band-like density in the right upper lobe. Linear densities at the bases.	Initial: ceftriaxone, azithromycin. Later: Levofloxacin, hydroxychloroquine.	Alive

46	M	None	Seizure, cough, fever, vomiting, diarrhea, passing out	Temperature 99.1 Fahrenheit reaching 102.9 Fahrenheit	Unremarkable	Sodium 131 mmol/L, creatinine 1.9 mg/dl, aspartate transaminase 62 U/L, alanine transaminase 73 U/L, alkaline phosphatase 225 U/L, creatine phosphokinase 1427 U/L	Clear lungs	Initial: Vancomycin, ceftriaxone, and acyclovir. Later: Azithromycin and hydroxychloroquine.	Alive

43	M	None	Shortness of breath, cough, fever, chest pain, headache	Temperature 97.6 Fahrenheit then 101.2 Fahrenheit	Breath sounds equal bilaterally	White count 2.1 k/*μ*L, hemoglobin 12.8 g/dl, platelets 110 k/*μ*L, erythrocyte sedimentation rate 36	Mild fullness in the right perihilar region	Levofloxacin but received hydroxychloroquine for two days.	Alive and discharged home

Key: age in years; M: male.
